# Targeting for Success: Demonstrating Proof-of-Concept with Mechanistic Early Phase Clinical Pharmacology Studies for Disease-Modification in Neurodegenerative Disorders

**DOI:** 10.3390/ijms22041615

**Published:** 2021-02-05

**Authors:** Maurits F. J. M. Vissers, Jules A. A. C. Heuberger, Geert Jan Groeneveld

**Affiliations:** 1 Centre for Human Drug Research, Zernikedreef 8, 2333 CL Leiden, The Netherlands; JHeuberger@chdr.nl (J.A.A.C.H.); GGroeneveld@chdr.nl (G.J.G.); 2 Leiden University Medical Center, Albinusdreef 2, 2333 ZA Leiden, The Netherlands

**Keywords:** clinical pharmacology, neurodegenerative disorders, disease-modification, proof-of-concept, mechanistic, phase 1 trials

## Abstract

The clinical failure rate for disease-modifying treatments (DMTs) that slow or stop disease progression has been nearly 100% for the major neurodegenerative disorders (NDDs), with many compounds failing in expensive and time-consuming phase 2 and 3 trials for lack of efficacy. Here, we critically review the use of pharmacological and mechanistic biomarkers in early phase clinical trials of DMTs in NDDs, and propose a roadmap for providing early proof-of-concept to increase R&D productivity in this field of high unmet medical need. A literature search was performed on published early phase clinical trials aimed at the evaluation of NDD DMT compounds using MESH terms in PubMed. Publications were selected that reported an early phase clinical trial with NDD DMT compounds between 2010 and November 2020. Attention was given to the reported use of pharmacodynamic (mechanistic and physiological response) biomarkers. A total of 121 early phase clinical trials were identified, of which 89 trials (74%) incorporated one or multiple pharmacodynamic biomarkers. However, only 65 trials (54%) used mechanistic (target occupancy or activation) biomarkers to demonstrate target engagement in humans. The most important categories of early phase mechanistic and response biomarkers are discussed and a roadmap for incorporation of a robust biomarker strategy for early phase NDD DMT clinical trials is proposed. As our understanding of NDDs is improving, there is a rise in potentially disease-modifying treatments being brought to the clinic. Further increasing the rational use of mechanistic biomarkers in early phase trials for these (targeted) therapies can increase R&D productivity with a quick win/fast fail approach in an area that has seen a nearly 100% failure rate to date.

## 1. Introduction

While there have been successes in neuropharmacology, most central nervous system (CNS) pharmaceutical approaches treat symptoms rather than disease cause. Such symptomatic treatments can be very successful at suppressing disease symptoms at first, however, the effects eventually diminish over time and do not stop disease progression. Therefore, there is an urgent need for better treatments that can slow or stop disease progression of neurodegenerative disorders (NDDs), especially since the burden of these debilitating diseases on patients and society is on the rise as populations age [1]. Alarmingly, the clinical failure rate for such disease-modifying treatments (DMTs) for NDDs has been nearly 100% to date [2,3,4,5]. Exceptions include the approval of riluzole and edaravone as treatments for amyotrophic lateral sclerosis (ALS); however, both arguably show only marginal effects [6,7]. With the recent approval of nusinersen for the treatment of spinal muscular atrophy (SMA) [8], new hope may be on the horizon.

In fact, our understanding of underlying NDD pathophysiological mechanisms is rapidly expanding [9,10,11,12,13], and this has sparked a new interest in the development of (targeted) disease-modifying treatments. This is reflected for example, by the >100 compounds currently in clinical development for Alzheimer’s disease [4] and close to 150 compounds in clinical development for Parkinson’s disease [14], many of which can be categorized as DMTs.

Compared to most other fields, the clinical development path of NDD DMTs faces some important additional challenges that contribute to the high failure rate experienced to date. First, preclinical and animal models have historically shown poor translatability to predict drug efficacy in human NDDs because of the complexity of the pathophysiology of neurodegenerative disorders and our incomplete understanding of these processes [2,15,16]. Secondly, in NDDs, it may take a long time from disease onset to the manifestation of clinical symptoms to objectifiable disease progression and clinical trials have struggled to separate out symptomatic effects from disease-modifying effects [2,16,17]. Moreover, by the time of diagnosis, significant (irreversible) damage to the CNS has often already occurred, and it has been challenging to identify robust diagnostic biomarkers to initiate treatment in earlier disease stages [18]. Thirdly, unlike diseases of most other organ systems, CNS disorders are localized to a body compartment that is not easily accessible for obtaining tissue samples in clinical studies to verify molecular pathophysiologic mechanisms and drug effects. Finally, there has been a lack of validated biomarkers as outcome measures for disease progression in disease-modification trials [16].

However, considerable progress is being made in the development of biomarkers for NDDs [19,20] that cannot only help diagnose or track progression of NDDs, but can also be used as tools during clinical development to demonstrate central exposure, (peripheral) target engagement and functional responses to guide dosing-decisions or facilitate patient enrichment in later stage clinical trials [21]. In particular, peripheral biomarkers for their relatively easy clinical accessibility hold a promise to help overcome some of the fundamental challenges in CNS drug development and allow for more efficient screening of drug candidates in early-phase clinical trials [22]. In a field where nearly 100% of investigational drugs fail to make it to market, the use of such biomarkers can offer an indirect yet relatively quick strategy to confirm (peripheral) target and pathway-engagement and provide early proof-of-concept in short-duration mechanistic early-phase trials in both healthy volunteers and patients [23,24]. This quick win/fast fail approach can increase research and development (R&D) productivity and help guide dosing-decisions for maximizing success rates in later stage trials [25].

Here we present a review and a roadmap for the use of pharmacodynamic biomarkers in early phase clinical trials of DMTs in NDDs. First, we present an introduction on NDD mechanisms, considerations for drug development of innovative disease modifying compounds, and the role of biomarkers in clinical drug development for context. Then we categorize the pharmacodynamic biomarkers that were reported in early phase clinical pharmacology studies identified from a literature review of the past decade, including an overview of bodily sources that can be used for biomarker analysis, and present considerations for biomarker selection in early clinical development. Finally, we summarize and conclude this overview with a proposal for a roadmap for designing mechanistic, data-rich early phase clinical pharmacology studies for disease-modifying therapies in neurodegenerative disorders.

## 2. Neurodegenerative Disease Mechanisms

Neurodegenerative disorders, including as Alzheimer’s disease (AD), frontotemporal- (FTD) and Lewy body dementia (LBD), amyotrophic lateral sclerosis (ALS), Huntington’s disease (HD), Parkinson’s disease (PD), and spinocerebellar ataxias (SCAs), are characterized by a progressive degeneration of neurons in various regions of the brain and result in losses in cognitive and/or motor function [26,27]. As it appears, these NDDs share multiple overlapping pathological mechanisms including misfolding, aggregation, and accumulation of proteins, dysfunctional mitochondrial homeostasis, formation of stress granules, and maladaptive innate immune responses, eventually leading to cellular dysfunction, loss of synaptic connections, and brain damage [28,29]. In AD, amyloid-β protein fragments that cluster together and form amyloid plaques, as well as tau proteins forming neurofibrillary tangles, disrupt neurological functioning and contribute to neurotoxicity leading to inflammation and neuronal cell death. In PD, clumping of α-synuclein into so-called Lewy bodies in dopaminergic neurons is believed to play an important role in neuroinflammation and eventually neurodegeneration, while in ALS, the aggregation of TAR DNA-binding protein 43 (TDP-43) in cell stress granules may contribute to disease pathology, neuroinflammation, and motor neuron death. Because of an overlap in the underlying pathological mechanisms, as well as involvement of the same cell types, it is not surprising that many DMT mechanisms under development often target multiple NDDs. For example, inhibition of receptor-interacting serine/threonine-protein kinase 1 (RIPK1), a regulator of inflammation, cytokine release, and necroptotic cell death, is being investigated as treatment for AD, ALS, and multiple sclerosis (MS) [30], while tau protein is being targeted with antibodies for both progressive supranuclear palsy (PSP) and AD [31]. In addition to the more general mechanisms of neurodegeneration, genetic studies have begun identifying risk-associated alleles and disease-causing rare mutations in NDDs [13,32]. These genetic studies may pave the way for targeted therapies in selected subpopulations, such as an antisense oligonucleotide targeting the mutated superoxide dismutase (SOD1) enzyme in ALS [33], or glucocerebrosidase (GBA)-activators or leucine-rich repeat kinase 2 (LRRK2)-inhibitors targeting disease-causing mutations in GBA or LRRK2 respectively in Parkinson’s disease [34].

## 3. Innovative Drug Development of Disease Modifying Treatments

The development of innovative disease modifying treatments for these NDDs with novel mechanisms of action is radically different from the development of a generic version of an existing effective drug from a well-established class [25]. For innovative compounds, the uncertainty about the different aspects of the drug is far greater, which is also reflected in the high clinical failure rate in the field of DMTs for NDDs. This uncertainty requires a high level of flexibility in the drug development program, the use of innovative methods, and a high level of integration of information rather than the purely operational requirements of a generic development program [25]. Innovative drug development in essence starts with the preclinical development of assays to identify and validate a novel pharmacological target, subsequently demonstrating safety and efficacy in a (relatively standardized) battery of laboratory and animal studies. Hereafter, the clinical development trajectory starts in humans and revolves around answering a set of six basic scientific questions in a series of what are traditionally called phase 1–3 clinical trials: (1) what is the safety and pharmacokinetic behavior of the drug, (2) does the drug occupy the intended pharmacological target, (3) is the drug capable of activating the target, (4) does this target activation lead to the intended physiological response, (5) and subsequently to the intended pathophysiological response, and (6) does the drug result in a sufficient clinical response [25]? Traditionally these questions are addressed in a chronological order, starting with small-scale phase 1 clinical studies focusing on safety and pharmacokinetics in healthy volunteers or patients and ending with large-scale, often global and multi-center, phase 3 studies to demonstrate safety and efficacy versus placebo or an active comparator in the intended drug label target population. However, as stated above, drug development does not need to take this linear approach. Especially if one considers that development becomes more and more expensive the further a compound progresses into later stage trails. In fact, for truly innovative compounds such as the development of DMTs in NDDs, there is a strong scientific and financial argument to be made to demonstrate proof-of-concept for a new compound in humans as early as possible [35]. From a scientific perspective, an early demonstration of proof-of-concept helps focus future efforts to the most promising leads. From a financial perspective, early proof-of-concept contributes to a quick win/fast fail development approach, thereby increasing R&D productivity and preventing investments in compounds only to fail in the most expensive later stages of drug development.

Demonstrating proof-of-concept of DMTs in early-stage trials is challenging, however. Considering the definition of a neurodegenerative DMT: “an intervention that produces an enduring change in the clinical progression of the NDD by interfering in the underlying pathophysiological mechanisms of the disease process leading to cell death” [36], proof-of-concept for the first part of this definition is difficult to demonstrate because of the short-duration of early phase clinical trials. Moreover, traditional clinical outcomes—such as disease progression scales or patient-reported outcomes (PROs)—are not suitable for demonstrating effects of DMTs in NDDs in healthy subjects for a lack of disease, nor in patients because of the general short duration and small group sizes in phase 1 trials and large placebo-effects in PROs often seen in these patient populations. The ability of an investigational compound to “interfere in the underlying pathophysiological mechanisms leading to cell death” on the other hand, is something that could be demonstrated with the use of pharmacodynamic biomarkers in short-duration early phase trials, even in healthy subjects.

## 4. Biomarkers

A biomarker (biological marker) is defined as “a characteristic that is objectively measured and evaluated as an indicator of normal biological processes, pathogenic processes, or pharmacological responses to a therapeutic intervention” [37]. When the level of a biomarker changes in response to exposure to a medical product, it can be called a *response or pharmacodynamic biomarker* [38]. Other types of biomarkers can include *diagnostic biomarkers* (detecting or confirming the presence of a disease), *predictive biomarkers* (presence or change in the biomarker predicts an individual or group to experience a favorable or unfavorable effect from the exposure to a medical product), *prognostic biomarkers* (identify the likelihood of a clinical event, disease recurrence, or disease progression in untreated patients), and *safety biomarkers* (indicates the likelihood, presence, or extent of a toxicity as an adverse event) [38,39]—see Table 1. In some cases, a biomarker can be used as surrogate to substitute for a clinical endpoint, but to qualify as a surrogate, a biomarker must correlate with the clinical outcome and the change in the biomarker must also explain the change in the clinical outcome [38]; evidence that is currently lacking for the majority of biomarkers.

Recent reviews have described the current status of biomarkers in ALS [40], Alzheimer’s disease [41], Parkinson’s disease [42], Huntington’s disease [43], and spinocerebellar ataxias [44], although for most of these indications, reliable indicators of disease severity, progression, and phenotype are still lacking.

## 5. Early Phase Proof-of-Concept with Mechanistic Biomarkers

Even without a proven correlation with clinical outcome, biomarkers are useful in early phase trials of DMTs for NDDs. At this stage of development, it is more important and feasible to demonstrate that the investigational drug engages its molecular pathway in humans as envisioned (mechanistic proof-of-concept). This can be accomplished with mechanistic biomarkers, by demonstrating pharmacologic activity of the compound both in healthy subjects as well as patients, allow for the application of mechanism-based pharmacokinetic/pharmacodynamic (PK/PD) modelling [46], and help define the optimal dose for phase 2/3 efficacy trials. This maximizes the eventual chance of clinical development success, or can save valuable resources by supporting an early “no-go” decision in case the compound fails to reach or appropriately modulate its target [21,47]. In fact, disease specific regulatory guidance for drug development in NDDs also recommends the use of biomarkers in the early phases of the clinical development to: (1) establish the pharmacological mechanism(s) on which the drug may be thought to have therapeutic activity, (2) demonstrate target engagement and proof-of-concept, and (3) determine the PK/PD relationship and the dose-response curve [48,49,50].

Additionally, by including a pharmacological effect or target engagement biomarker in a first-in-human (FIH) study, the dose-response curve in humans can be linked to the non-clinical experience, thereby supporting more informed dose escalation decisions. This is especially true for innovative drugs with a novel mode of action, where the relationship between the minimally pharmacologically active dose and a safe therapeutic dose in humans is not yet known [51]. Inclusion of a pharmacodynamic measure in FIH trials is now also recommended by the regulatory bodies for safety reasons [52].

## 6. Reported Use and Classification of Early Clinical Phase Biomarkers

As indicated above, biomarkers can play an important role in early phase drug development. To investigate the current use of pharmacodynamic response biomarkers for the development of DMTs for NDDs, a literature search was performed for published early phase clinical trials using medical subject headings (MESH) terms in PubMed (Supplement Material). Publications between 2010 and November 2020 were selected that reported an early phase clinical trial with NDD DMT compounds. Publications of early phase trials identified from references in the reviewed literature that were not identified by the MESH search strategy were also included. Only the first and original reports of early phase clinical trials were selected to avoid duplication (Figure S1). An overview of all included trials and the reported peripheral and central pharmacodynamic biomarkers is presented in Table 2.

The early clinical phase pharmacodynamic response biomarkers retrieved from this search can be subdivided into *proximal* mechanistic biomarkers that are primarily used to demonstrate *target occupancy* and *target activation* (target engagement), and *physiological* and *pathophysiological response* (distal) biomarkers (Table 1) [25,46]. 

**Figure 1:**
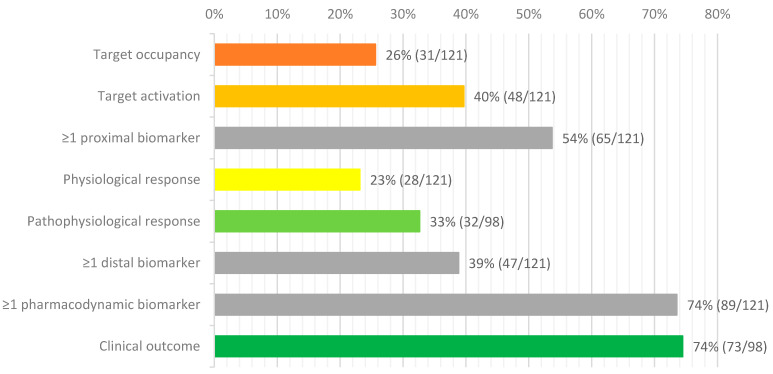
Percentage of early clinical phase reporting the use of different categories of pharmacodynamic biomarkers and clinical outcomes. Thirty-one trials (26%) reported the use of target occupancy biomarkers and forty-eight trials (40%) reported the use of a target activation biomarkers. Sixty-five trials included at least 1 proximal (mechanistic) biomarker (target occupancy and/or activation). Twenty-eight trials (23%) reported the use of physiological response biomarkers. Thirty-two trials used pathophysiological response biomarkers, which comes down to 33% of all early phase NDD DMT trials (98) that were performed in patients. Forty-seven trials (39%) reported the use of at least 1 distal biomarker. In total, 89 of 121 trials reported at least one pharmacodynamic biomarker and seventy-three trials reported clinical outcomes, which comes down to 74% of all early phase NDD DMT trials (98) that were performed in patients.

Overall, 89 out of 121 (74%) NDD DMT early phase trials that were published over the past decade reported the use of one or more pharmacodynamic response biomarkers (Figure 1). Given the significant added value of using pharmacodynamic response biomarkers in early phase trials, this might not be surprising. Less than half of all trials (46%) reported the use of central pharmacodynamic biomarkers. The use of peripheral pharmacodynamic biomarkers was slightly higher at 50%. Only 65 trials (54%) reported the use of proximal mechanistic biomarkers (Figure 1) and there are clear differences in the use of biomarkers between different disorders and different types of drugs (Table 2).

Clinical outcome data was collected even more frequently in early clinical phase NDD trials (74% of all trials involving patients, or 60% of all trials) than mechanistic biomarker read-outs (54% of all trials) (Figure 1). This despite the fact that early phase trials are often of too short a duration and have a too limited sample size to expect a significant effect on any clinical or surrogate response biomarkers.

**Figure 2:**
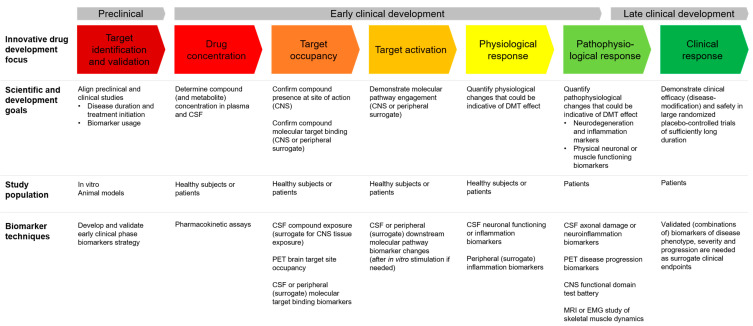
Roadmap for early phase clinical development of disease-modification therapies in neurodegenerative disorders, focusing on demonstrating proof-of-concept with mechanistic early phase clinical pharmacology studies. Innovative clinical drug development revolves around confirming the pharmacokinetic behavior of the drug, occupation, and activation of the intended pharmacological target in humans, quantifying the subsequent physiological and pathophysiological response before moving into large late stage trials to demonstrate a clinical response (long-term disease modification). Safety evaluation is not specifically mentioned but is obviously an essential component at each stage of clinical drug development. For each stage of drug development, different biomarker techniques can be used to come to an early mechanistic proof-of-concept, define the optimum dose, and facilitate a validated “go/no-go” decision before moving into expensive late stage trials.

In the next sections, we will break down the different types of identified biomarkers. For each stage of drug development, these different types of biomarkers can help answer different relevant clinical development questions; see also Figure 2. 

### 6.1. Target Occupancy

Only 26% of early clinical phase NDD DMT trials reported target occupancy biomarkers (Figure 1 and Table 2). Target occupancy in first-in-human studies is used to demonstrate that the same target binding observed in the preclinical animal models holds true in humans [171]. The importance of this from a safety perspective is exemplified by the clinical study with the CD28 targeting immunomodulating agent, TGN1412. Because of differences in TGN1412 pharmacology between nonhuman primates and humans, the starting dose of the FIH trial directly resulted in 90% receptor occupancy, leading to life-threatening cytokine release syndrome in healthy volunteers [172,173].

Demonstrating target engagement is also critical from the drug-development perspective. When a novel compound fails to demonstrate disease-modifying properties and no target engagement data is available, it will be difficult if not impossible to conclude whether the mechanism of action does not produce NDD disease-modification per se, or if this specific compound was just not successful in sufficiently engaging the intended target in humans [174,175].

Ideally target occupancy is demonstrated by biomarker evidence of (1) the compound reaching its site of action, (2) the compound binding to the intended molecular target, and (3) occupancy of the target increases with increasing dose.

Demonstrating that a compound reaches its site of action is one of the major challenges in CNS drug development, and in fact often not even possible to demonstrate directly (except post-mortem). As an alternative, often the presence of the compound at pharmacologically active concentrations in the cerebrospinal fluid (CSF) is used as a surrogate for CNS exposure [2,23,30,54]. While this is not an absolute guarantee that the compound reaches its site of action in the brain, it does provide a relatively uncomplicated method (it can even safely be used in pediatrics [176]) to demonstrate that the compound does cross the blood-brain barrier in sufficient concentrations to expect an effect based on preclinical cellular dose-response assays. In addition, further translational approaches can be used to predict human brain distribution and target site kinetics [177].

Besides measuring compound concentration in CSF, positron emission tomography (PET) can be used to demonstrate compound distribution into specific brain compartments and can in some cases also be used as a direct occupancy assay for receptor, transporter or enzyme targets [178,179]. However, PET imaging cannot always be applied for the lack of an appropriate radioligand or unfavorable radioligand characteristics, e.g., high non-specific binding [159].

Actual binding of the compound to the molecular target could in some cases be demonstrated in the CSF, for example for monoclonal antibodies binding to a circulating extracellular target protein such as amyloid β [54,55,56,60] or α-synuclein [146] (Table 2). However, this may not always be possible because assays are either not sensitive enough to detect the low abundance pathological target (e.g., aggregated α-syn concentrations in CSF) or drug concentrations in the CSF are not sufficient to demonstrate an effect on a more abundant surrogate biomarker (e.g., total α-syn in CSF) [145].

For (intra)cellular targets in CNS tissue, it may be even more difficult to demonstrate that the compound binds the intended molecular target, mainly because of the fact that these cellular molecules are likely not present in biofluids in detectable amounts and the target neuronal cells cannot be sampled from living human beings for cell lysis and subsequent target engagement assays. In these cases, an alternative indirect strategy could be to demonstrate target engagement in peripheral cells, on the condition that the molecular target is expressed in these cells. For example, peripheral receptor occupancy on cell surfaces can be measured with the use of flow cytometry on fresh blood [180]. In a similar fashion, intracellular target occupancy can be demonstrated peripherally in blood cells such as done for LRRK2-inhibitor binding measured via the dephosphorylation of Ser935 on the LRRK2 protein in lymphoblastoid cells [181], or the reduction of phosphorylated S166 RIPK1 in peripheral blood mononuclear cells (PBMCs) after dosing of an RIPK1-inihibitor [30]. When combined with the plasma-to-CSF drug concentration ratio, such peripheral target occupancy can give an indirect indication of expected target occupancy in the CNS.

### 6.2. Target Activation

After confirming that a novel compound occupies its molecular target, the next step in innovative clinical development is to demonstrate that upon target occupation, the investigational compound activates the intended molecular pathway to a sufficient extent for possible disease modification (Figure 2). Such mechanistic proof-of-concept can often be demonstrated by evaluating a substrate biomarker that is downstream in the pathway of the compound’s direct molecular target. When quantitatively measured, changes in such a so-called ‘pathway activation biomarker’ at different dose-levels can help generate a dose-response curve of the investigational compound’s agonistic (stimulatory or inhibitory) molecular effects. This dose-response curve can be linked to the preclinical in vitro and animal model studies to determine a human dose level at which maximum disease modification can be expected in patients. Target activation biomarkers have been used more frequently than target occupation biomarkers, but still only 40% of early clinical phase NDD DMT trials reports the use of target activation biomarkers (Figure 1).

An example of a molecular pathway activation biomarker is the quantification of amyloid β_1–42_ (Aβ) concentrations in the CSF in response to BACE1-inhibitors [84,85,86,87,88,89,90] (Table 2). BACE1 (β secretase) is a protease that cleaves the amyloid precursor protein at the β-site, which eventually leads to the production and release of Aβ peptide in the brain. A decrease in Aβ brain concentrations may help prevent the progression of Alzheimer’s disease [182]. However, as indicated before, such an apparently obvious relationship between the molecular pathway activation biomarker to the neurodegenerative disease that the compound is being developed is not a necessity. It is more important that the biomarker has a direct relationship to the true molecular target that the investigational compound activates or inhibits, and that the biomarker can reliably be measured with a robust and validated assay. An example is the quantification of phosphorylation of Rab10 (pRab10), a bona-fide substrate of LRRK2 kinase activity, in response to the administration of LRRK2-inhibitors under development for Parkinson’s disease [183]. The fact that at the time of discovery it was not entirely clear how the activity of Rab GTPases contributes to degeneration of the nervous system [184] does not impact the usability of pRab10 as target activation biomarker to quantify the inhibitory effects of LRRK2-inhibitors.

Similar to target occupancy, it may not always be possible to demonstrate target activation in the CNS, especially for intracellular molecular pathways, in which case an alternative strategy can also be to demonstrate target activation peripherally in blood or tissues expressing the same molecular target [120,126,127,130] (Figure 2).

Demonstrating target activation can be complicated by the fact that the targeted molecular pathway activation status may only be present in diseased tissue. For example, RIP kinase 1 regulates inflammation, cytokine release, and necroptotic cell death and inhibition of RIPK1 activity protects against inflammation and cell death in multiple animal models. RIPK1 is also expressed in circulating PBMCs offering a peripheral opportunity to demonstrated target activation of RIPK1-inhibitors. However, in these non-diseased PBMCs, RIPK1 activity levels will not be similar to that in the CNS of ALS and AD patients. To overcome this problem and quantify the effects of different dose levels of a RIPK1-inhibitor peripherally, PBMCs can be collected from study subjects after dosing and then be stimulated in vitro with e.g., the pan-caspase inhibitor zVAD- FMK (TSZ) to stimulate these cells to increase phosphorylated RIPK1 [30]. In a similar fashion, lipopolysaccharide (LPS) has been used in an early phase study in MS patients to stimulate 6-sulpho LacNAc+ dendritic cells in vitro, to demonstrate that laquinimod therapy is capable of reducing CD83 expression and TNF-α production [138]. The possibility to demonstrate target activation in vitro in human cells is supported by regulatory guidance [50], and could be used to demonstrate target activation in first-in-human studies with healthy volunteers [30]. Some molecular targets are really only present in patients with the target disease, such as mutated huntingtin protein in patients with Huntington’s disease. In such a case, the best strategy may therefore be to directly include patients in the earliest clinical trials, to be able to demonstrate target activation as early as possible in the clinical development trajectory [131].

Other types of target activation biomarkers may be used for different classes of investigational drugs (see Table 2). For example, in the case of immunotherapy, target activation could be demonstrated by the formation of antibody titers in plasma [156], and in the case of an antisense oligonucleotide, target activation may be demonstrated by a reduction in target protein levels [33,167]. For other types of drugs such as monoclonal antibodies against amyloid β [53,54,55,56,57,58,59,60,61,62,63], it may not be possible to demonstrate target activation, as the goal of these treatments is to clear the molecular target either by macrophage phagocytosis and complement activation or by altering the equilibrium of amyloid across the blood–brain barrier in favor of efflux from the brain to the blood [185].

### 6.3. Physiological Response

Physiological response biomarkers are reported in 23% of early phase NDD DMT clinical trials (Figure 1). These provide insight into more general or systemic (distal) responses to the investigational compound that are expected to contribute to, or be indicative of, possible disease modification. Examples of physiological response markers that have been used in early phase NDD DMT clinical trials include the evaluation of brain glucose metabolism after administration of nerve growth factor gene therapy [68] or deep brain stimulation [76,78] for Alzheimer’s disease, and CSF cytokine production after transfusion of stem cells [101] or administration of granulocyte colony-stimulating factor (G-CSF) [115] in ALS patients (see Table 2). However, it is important to realize that while such biomarkers can indicate that a compound exerts a physiological response, they often do not provide direct information about the actual clinical effects of the compound [25], nor that the intervention can produce an enduring change in the clinical progression of the NDD. Nevertheless, when combined with target occupancy and activation biomarkers, physiological response biomarkers can contribute to the total amount of evidence for proof-of-concept (see Figure 2). Additionally, physiological response markers can offer an opportunity to get a better understanding of an intervention’s potential effects when no direct molecular target is involved or when the exact mechanism of action is not yet fully understood, e.g., in the case of stem cell trials in ALS patients [101,104] (Table 2).

### 6.4. Pathophysiological Response

Pathophysiological response biomarkers are also distal biomarkers, and contrary to the physiological response biomarkers, should have a clear and direct link to the disease pathophysiological mechanisms. For early phase trials, these biomarkers do not necessarily need to be validated surrogate substitutes for clinical endpoints, however, when available, a validated surrogate would of course provide stronger evidence for possible disease modification. It should be considered though that most early phase trials are only of a short duration and for most NDDs the disease progresses too slow to measure a significant change over a short period of time. Moreover, early phase trials usually only recruit small sample sizes and there can be significant interindividual variation in disease phenotype and progression. Therefore, chances are that it may not be possible to demonstrate a significant effect of the investigational compound on pathophysiological response biomarkers in early phase trials, which would not necessarily equal a lack of effect of the investigational compound. It is therefore not surprising that pathophysiological response biomarkers are only reported in 33% of early phase clinical trials involving patients (Figure 1). In healthy volunteer studies, pathophysiological response biomarkers obviously cannot be included for a lack of disease presence.

Examples of pathophysiological response wet biomarkers that have been used in early phase NDD DMT trials include quantification of CSF tau phosphorylated at threonine 181 (*p*-tau181) [54,60] and evaluation of amyloid β by PET [75] for Alzheimer’s disease pathology, phosphorylated neurofilament heavy chains (and post-hoc neurofilament light chain) concentrations as general axonal damage biomarker in ALS [33], FTD [129], and Huntington’s disease [131], and CSF mitochondrial dysfunction markers (GDF15, lactate) in MS [139] (Table 2). Other types of more physical pathophysiological response biomarkers include the evaluation of retinal nerve fiber layer thinning in MS [139] and electromyogram (EMG) study of the tibialis anterior muscles in ALS patients receiving stem cell treatment [109]. In addition, neuroimaging techniques can be used as pathophysiological response biomarkers, such as the evaluation of disease progression via dopaminergic function with the use of 18F-dopa PET [153], or reduction of whole brain or hippocampal atrophy (MRI) or reduction of cerebral metabolism on fluordeoxyglucose (FDG) PET [36], although it is unlikely that an effect on these markers can be observed in short-duration trials.

### 6.5. Clinical Response

It appears that clinical outcomes are most frequently included (74%) as exploratory endpoints in early phase trials with NDD patients (Figure 1). These clinical outcome measures included disease rating scales (e.g., Alzheimer’s Disease Assessment Scale-Cognitive Subscale (ADAS-Cog) [53,70,73,78], Mini-Mental State Examination (MMSE) [58,61], Revised Amyotrophic Lateral Sclerosis Functional Rating Scale (ALSFRS-R) [33,106,119], Neuronal Ceroid Lipofuscinosis Type 2 (CLN2) Clinical Rating Scale [143], Unified Huntington’s Disease Rating Scale (UHDRS) [132], Hammersmith Functional Motor Scale Expanded (HFMSE) [167], and Movement Disorders Society Unified Parkinson Disease Rating Scale (MDS-UPDRS) [153,154,161]), pulmonary functioning evaluation [100,128] muscle power assessments [99,103,113], and quality of life questionnaires [68,120,152]. We would argue, however, that due to small samples sizes in early phase trials, potentially significant placebo effects or sometimes lack of a placebo control, and the relatively low sensitivity of these disease rating scales such instruments may at best be useful as safety biomarkers but not as outcome markers at this stage of clinical development. Even in longer-duration, open label extensions of early phase trials clinical outcomes are not expected to yield reliable results because of the small sample sizes and lack of a placebo control [186]. However, the high percentage of early phase trials reporting clinical outcomes may result from regulatory guidance that recommends to explore clinical outcomes in early phase trials to investigate how these can be further used in subsequent pivotal trials [49]. A more sensitive future tool for assessing exploratory clinical outcomes on disease progression could be the use of continuous digital biomarkers, such as smartphone-based assessments [187].

## 7. Biomarker Sources

Cerebrospinal fluid (31% of trials) and blood (45% of trials) are the most frequently used biofluids for biomarker analysis in NDD research. These biofluids are relatively easily accessible in the clinical setting and well-established bioanalytical methods for these matrices are available. CSF could arguably be the most proximal source for physiological and pathological response biomarkers related to the intended CNS target. Moreover, concentrations of CNS biomarkers outside of CSF are often extremely low, making them difficult to detect using standard assays, and in blood endogenous antibodies and proteases may be present that interfere with assays or shorten the lifespan of peripheral protein biomarkers [18]. However, as discussed previously, mechanistic proof-of-concept of target engagement by DMT compounds can often be demonstrated very well peripherally without being hampered much by such challenges. Moreover, NDDs are found to also be influencing some peripheral tissues outside the CNS [188]. Therefore, in early stage drug development, pharmacodynamic biomarkers can be used from a large variety of bodily sources (see Table 2). Besides whole blood, plasma or serum, leukocytes and in particular the subset of PBMCs can be an easily accessible source for evaluating intracellular pathways ex vivo, which also offers the possibility to simulate disease states (also in heathy volunteer studies). When working with PBMCs though, it is important to realize that these cells represent a heterogeneous group that includes lymphocytes, monocytes, and macrophages and the molecular target of interest may not be expressed to similar levels in all of these cells. For example, LRRK2 kinase and its direct substrate Rab10 are only abundantly expressed in monocytes and are virtually undetectable in B and T lymphocytes as well as natural killer and dendritic cells that constitute most of the PBMCs [189]. Moreover, both these proteins are expressed to an even higher degree in neutrophils, making neutrophils potentially the best source for demonstrating mechanistic proof-of-concept of LRRK2-inhibitors [189]. Another easily accessible biofluid that can be a source for biomarker analysis is urine [190], but also more challenging matrices, such as stool samples, ocular fluids, and mucosal secretions can be considered for biomarker analyses [191]. The challenge of accurate analysis, however, is much higher in such matrices and therefore feasibility of sampling as well as analyte extraction should be considered and demonstrated prior to implementation in clinical trials [191]. Furthermore, tissue biopsies, such as from muscle [99] or nasal olfactory neural tissue [192], and surgical byproducts [191] can be considered as sources for biomarker analysis. Even the body surface has proven to be an easily accessible source for biomarker analysis in NDD drug development via the use of skin fibroblasts [193] and hair follicle RNA [194].

As there may be relatively large intra- and interindividual variability in some of the biomarkers in these matrices, it could be necessary to normalize the biomarker read-outs to a quantifiable reference value to draw more robust conclusions between different sampling times and individuals. This is especially important given the small numbers of subjects usually included in early phase trials. Examples of normalization factors used in biomarker analysis include normalization to total protein or creatinine to correct for the number or concentration of cells in a specific sample or matrix for gene expression analysis [191], relating analysis of SOD1 activity in erythrocytes to the content of hemoglobin in erythrocyte lysates [120], relating phosphorylated glycogen synthase (GS) to the total levels of GS [129], and using the survival of motor neuron 2 full length (SMN2FL)/SMN2Δ7 mRNA ratio to reduce the confounding effects of SMN2FL and SMN2Δ7 mRNA level fluctuations for monitoring the inclusion of SMN2 exon 7 and the effect of risdiplam [169]. In addition, using patients as their own controls with cross-over designs in early phase clinical trials helps limit the potential effects of often large inter-subject variability in studies with small numbers of subjects [81]. Finally it can be worth considering using patient enrichment strategies for early phase trials [195], to optimize the chance of success in demonstrating proof-of-concept by including the most suitable patient population (e.g., with a specific genetic mutations, disease onset state, or a slow or fast disease progression prognosis). The scientific benefit of targeting a specific subpopulation, however, should be balanced to the recruitability of the trial and potentially the targeted mode of action.

## 8. Biomarker Selection, Development, and Validation

The decision to evaluate biomarkers in early phase clinical trials should be taken well in advance in order to select appropriate biomarkers to address the key scientific early phase clinical development questions and develop robust bioanalytical methods [25,191]. In fact, the biomarker strategy planning for first-in-human studies should ideally start during the preclinical development phase (Figure 2). Steps to consider when selecting biomarkers for use in early phase clinical trials include defining the scientific questions that the biomarker should help answer, performing a thorough literature review to select fit-for-purpose biomarker, bioanalytical method development or assay and laboratory selection, analytical model validation testing, and defining the clinical sampling, data reduction and analysis strategy [191,196]. Preferably the selected biomarkers are validated in the preclinical models used during drug development as well as in patients or patient biofluid repositories [197]. Characteristics to select a useful biomarker include that the biomarker should give a consistent response across studies and drugs with the same mode of action, must respond clearly to therapeutic doses, must have a clear dose-response relationship and ideally there should be a plausible relationship between the biomarker, pharmacology of the drug class, and disease pathophysiology (although for mechanistic biomarkers, this not an absolute necessity as discussed previously) [25].

Biomarkers used in early phase clinical development do not fall under standardized regulatory requirements and therefore the clinical development team has to decide on the level of method characterization and documentation that is needed by weighing how the biomarker may provide the most value to the clinical development program goals [191]. For an early go/no-go decision, a qualified assay may fit the purpose, whereas for proof-of-concept of clinical responses, a fully validated method may be required [191]. Some biomarkers used in early phase trials may evolve over time to become diagnostics or surrogate endpoints, but this requires the biomarkers to become accepted for use through submission of biomarker data during the drug approval process or via the biomarker qualification program developed by the Center for Drug Evaluation and Research [39].

## 9. Limitations

It is clear that the use of pharmacodynamic biomarkers in early phase clinical trials can help optimize clinical development in an area that has seen a near 100% failure rate to date, and that the frequency of rational use of these pharmacodynamic biomarkers should be improved (Figure 1). However, the use of pharmacodynamic biomarkers in itself is obviously not a guarantee for clinical development success. There are still some major challenges that the development of DMTs for NDDs faces that the use of biomarkers will not be able to solve.

DMT development has been struggling with a poor translatability of preclinical and animal models to human disease [15], though in the past decade, great advances have been with neurons derived from induced pluripotent stem cells (iPSCs) and 3D cell cultures technologies as pre-clinical models for neurodegenerative diseases [198]. While the use of biomarkers will not directly impact the quality of the animal models, biomarkers may help identify subsets of patients or early versus late stage disease states to better align the preclinical work with the target population for human proof of concept studies. Moreover, when preclinical and early stage clinical biomarker programs are well aligned, they can help demonstrate early proof-of-concept and translatability of target engagement in humans. Especially when combined with upcoming preclinical or translational PK/PD modeling and simulation (M&S) techniques [199], mechanistic biomarkers can in this way contribute to early ‘go/no-go’ development decisions and thereby help improve R&D productivity in the development of NDD DMTs.

Another challenge for the development of DMTs for NDDs is that our current disease understanding or hypotheses may be wrong, and that even when biomarkers demonstrate target engagement in humans, there may be no clinical disease-modifying effects of the compound [2]. However, in this case, it is essential that target engagement was demonstrated in the early phase trials, as this would point towards limited clinical relevance of the targeted pathway as a whole, rather than possibly just a lack of effect of the specific compound itself.

The usefulness of biomarkers must also not be overestimated. Early phase clinical trials may be of too short a duration to demonstrate an effect on disease progression biomarkers and therefore a lack of effect on a pathophysiological response marker in early phase trials does not necessarily mean that there can be no long-term clinical effect. Another caveat to be aware of is that treating a biomarker may not treat the disease, as has become clear in the development of anti-amyloid therapies. While anti-amyloid antibodies, BACE inhibitors, and γ-secretase inhibitors all demonstrated target engagement in early phase trials, they all subsequently failed to demonstrate clinical effect in later stage trials [200]. This could potentially indicate that targeting amyloid β may after all not contribute to disease modification in Alzheimer’s disease, or that amyloid β-targeting therapies need to be administered in a much earlier disease state for which we currently still lack robust diagnostic biomarkers.

Moreover, as no single one biomarker to date has been demonstrated to be indicative of NDD disease progression, it is recommended to use multiple response biomarkers when available to establish a pattern or fingerprint of treatment effects [201,202], contributing to the overall persuasiveness of proof-of-concept for a disease-modifying effect.

Finally, it should be kept in mind that developing a robust biomarker strategy can be a very lengthy and time-consuming process, and this process should therefore already be initiated well in advance of the first-in-human studies. This requires a strong collaborative effort between the preclinical scientists and the clinical development team to ensure a seamless integration of the preclinical and early-stage clinical biomarker strategies [25], which in the end might prove to be the most critical parameter for success in early stage NDD DMT development.

## 10. Roadmap for Mechanistic, Data-Rich Early Phase Clinical Pharmacology Studies

Over the past decade, the toolbox for early phase clinical development for NDDs has expanded significantly, which will hopefully help bring the first DMTs to patients in the decade to come. In AD (79%) and PD (71%), pharmacodynamic biomarkers by now have a well-established role in early clinical development, but in for example ALS (52%) and PSP (25%) there is still room for significant improvement (Table 2). In Figure 2, we therefore propose a best-practice roadmap for mechanistic, data-rich early phase clinical pharmacology studies for disease-modifying therapies in neurodegenerative disorders. Even if modifying the course of NDDs could ultimately prove to require a multi-drug approach, it will remain essential to clearly demonstrate pathway engagement of each individual drug component to get to rational multi-drug treatment regimens.

## 11. Conclusions

As our understanding of NDDs is improving, there is a rise in potentially disease-modifying treatments being brought to the clinic. Further increasing the rational use of mechanistic biomarkers in early phase trials for these (targeted) therapies can increase R&D productivity with a quick win/fast fail approach in an area that has seen a nearly 100% failure rate to date.

## Figures and Tables

**Table 1:** Biomarker categories and examples of use in NND DMT drug development (adapted from Cummings and Amur et al. [39,45]).

Biomarker Category	Use in Drug Development	Examples from NND DMT Drug Development
Response	Pharmacodynamic biomarker as indicator of intended drug activity	CSF total amyloid-β and fragments in response to amyloid-β antibody treatments
	*Proximal* (molecular target occupancy and activation) *Distal* ([patho]physiological response)	
	Efficacy response marker as a surrogate for a clinical endpoint	Braak staging with tau PET as a surrogate biomarker for clinical AD (though no validated surrogate biomarkers are available yet for NDDs).
Diagnostic	Patient selection	GBA1 gene mutation in PD patients SOD1 gene mutation in ALS patients
Predictive	Patient stratification Trial enrichment via inclusion criteria	Tau PET to identify AD patients more likely to respond to anti-tau therapies
Prognostic	Patient stratification Trial enrichment with patients likely to have disease	Percentage of weight loss at baseline for life expectancy and disease progression in ALS patients
Safety	Detect AEs and off-target drug responses	MRI for structural changes (including tumor or syrinx formation) within the brain after stem cell transplantation for ALS

**Table 2:** Overview of published early phase clinical trials for disease-modifying compounds in neurodegenerative disorders between 2010 and November 2020 and reported peripheral and central pharmacodynamic biomarker outcomes.

Indication	Drug Category	Drug Target	Trials Reporting Mechanistic Biomarker	Peripheral Biomarkers	Central Biomarkers	Types of Biomarkers	Study Population	References
AD	Antibody	Amyloid β	10/11 (91%)	Plasma total Aβ and Aβ fragments (Aβ1-x, Aβ1-40, Aβ1-42, Aβ3–42, Aβ1–38, Aβ18–35)	CSF Aβ species (Aβ1-x, Aβ1-40, Aβ1-42), t-tau, and p-tau181	Target occupancy and pathophysiological response	HVs and patients	[53,54,55,56,57,58,59,60,61,62,63]
		Tau protein	1/1 (100%)	-	CSF N-terminal tau, mid-domain tau, Aβ40, and Aβ42	Target occupancy and physiological response	HVs	[64]
	Cell therapy	Cytotropic factors, anti-inflammatory, neurogenesis	1/1 (100%)	-	CSF Aβ, t-tau and p-tau; PiB-PET changes in parenchymal amyloid deposition; FDG-PET metabolic changes	(patho)physiological response	Patients	[65]
		Nerve growth factor	0/1 (0%)	-	-	-	Patients	[66]
	Dietary	Xanthophyll Carotenoids, Omega-3 Fatty Acids	0/1 (0%)	-	-	-	Patients	[67]
	Gene therapy	Nerve growth factor	1/1 (100%)	-	PET brain glucose metabolism (post-mortem brain autopsy gene-mediated NGF expression and bioactivity)	Physiological response (and post-mortem target occupancy and activation)	Patients	[68]
	Growth factor	Nerve growth factor	1/1 (100%)	-	MRI for implant position; CSF Aβ1–42, t-tau, p-tau181, NfL, glial fibrillary acidic protein (GFAP), AChE and choline acetyltransferase (ChAT) activity and protein levels	Target occupancy, activation and (patho)physiological response	Patients	[69]
	Immunotherapy	Amyloid β	3/3 (100%)	Plasma anti-Aβ40 antibodies, Aβ peptides (Aβ40, Aβ42) and cytokines (IL-6, TNF-α, IL-1β, MCP-1, IL-2, sIL-2R); Serum antibody titres (Aβ IgM, Aβ IgG), Aβ1–40, AβX–40, Aβ1–42; In Vitro lymphocyte proliferation and cytokine production; PBMC β-specific and Qβ-specific responses of T-cells	CSF antibody titres, AβX–40, AβX–42, Aβ1–42, AβN–42, t-tau, p-tau181; MRI brain volumetric assessment	Target activation and (patho)physiological response	Patients	[70,71,72]
		Tau protein	1/1 (100%)	IgG and IgM titre anti-vaccin peptide, anti-KLH antibody titre, anti-pathological-tau antibody titre; Lymphocyte immunoprofiling	-	Target activation and physiological response	Patients	[73]
	Peptide	Amyloid β	0/1 (0%)	-	-	-	HVs	[74]
	Focused ultrasound with injected microbubbles	BBB-opening to amyloid β and tau	1/1 (100%)	-	PET BBB opening and amyloid β deposition	Target occupancy and pathophysiological response	Patients	[75]
	DBS	Cerebral glucose metabolism	3/4 (75%)	-	PET cerebral glucose metabolism	Physiological response	Patients	[76,77,78,79]
	Small molecule	5-HT2A receptor	0/1 (0%)	-	-	-	HVs	[80]
		Amyloid precursor protein (APP) synthesis	1/1 (100%)	-	CSF sAPPα, sAPPβ, t-tau, p-tau, Aβ42 and inflammatory markers (complement 3, factor H, MCP-1, YKL-40, sCD14)	Target activation and (patho)physiological response	Patients	[81]
		Amyloid production and associated inflammatory response	0/1 (0%)	-	-	-	HVs	[82]
		BACE1	7/8 (89%)	Plasma total Aβ and Aβ fragments (Aβ1–37, Aβ1–38, Aβ1–40, Aβ1–42, Aβx-40), total sAPP and fragments (sAPPα, sAPPβ)	CSF total Aβ and fragments (Aβx-38, Aβx-40, Aβx-42, Aβ1–37, Aβ1–38, Aβ1–40, Aβ1–42), total sAPP and fragments (sAPPα, sAPPβ), BACE1, t-tau, p-tau181	Target occupancy, activation and pathophysiological response	HVs and patients	[83,84,85,86,87,88,89,90]
		ET(B) receptor	0/1 (0%)	-	-	-	HVs	[91]
		Glutaminyl cyclase (QC)	1/1 (100%)	Serum QC activity	CSF QC activity	Target occupancy and activation	HVs	[92]
		Glycogen synthase kinase-3β (GSK3β)	1/1 (100%)	Lymphocyte GS phosphorylation	-	Target occupancy	HVs	[93]
		Sigma-2 receptor complex	0/1 (0%)	-	-	-	HVs	[94]
		γ-secretase	2/2 (100%)	Plasma Aβx–42	CSF total Aβ and Aβ fragments (Aβ42, Aβ40, Aβ37, Aβ38)	Target activation	HVs	[95,96]
		RIPK1 inhibitor *	1/1 (100%)	PBMCs reduction of pS166 RIPK1	-	Target occupancy and activation	HVs	[30]
		Microtubule stabilization	1/1 (100%)	-	CSF NfL, t-tau, p-tau, Aβ42, YKL-40	Pathophysiological response	Patients	[97]
	Cell therapy	Neuroprotective effects	1/1 (100%)	-	CSF t-tau, p-tau, Aß42	Pathophysiological response	Patients	[98]
Overall use of mechanistic biomarkers in early phase AD trials			37/47 (79%)					
ALS	Antibody	Neurite outgrowth inhibitor Nogo-A	1/1 (100%)	Muscle biopsy Nogo-A RNA and protein expression; Plasma Nogo-A protein gamma sarcoglycan; EMG (MUNE)	-	Target occupancy and activation	Patients	[99]
	Antisense Oligonucleotide	SOD1	2/2 (100%)	Plasma *p*-NfH, NfL	CSF SOD1, *p*-NfH, NfL	Target activation and pathophysiological response	Patients	[33,100]
	Cell therapy	Neurotrophic growth factors and cytokines secretion, immunomodulation and cell proliferation or replacement	5/13 (38%)	MRI muscle volume CD4 + CD25 + FOXP3 + Tregs, proliferation of autologous responder T lymphocytes; EMG of TA muscles (CMAP, FD, SMUP, MUNE, MUNIX, MUSIX); EIM	CSF cytokines (TGF-b1, TGF-b2, TGF-b3, IL-6, IL-10, MCP-1)	(patho)physiological response	Patients	[101,102,103,104,105,106,107,108,109,110,111,112,113]
	Gene therapy	Hepatocyte growth factor	1/1 (100%)	Serum HGF; Muscle circumference	-	Target activation and pathophysiological response	Patients	[114]
	Growth factor	Granulocyte colony-stimulating factor	1/1 (100%)	Blood cell counts, CD34 + cells, serum cytokines/chemokines (IL-1b, IL-1ra, IL-2, IL-4, IL-5, IL-6, IL-7, IL-8, IL-9, IL-10, IL-12 (p70), IL-13, IL-15, IL-17, eotaxin, bFGF, FGF-2, TGF-a, G-CSF, GM-CSF, IFN-γ, IP-10, MCP-1, MIP- 1a, MIP-1b, PDGF-BB, RANTES, TNF-a, VEGF)	CSF BMC presence, cytokines/chemokines (IL-1b, IL-1ra, IL-2, IL-4, IL-5, IL-6, IL-7, IL-8, IL-9, IL-10, IL-12 (p70), IL-13, IL-15, IL-17, eotaxin, bFGF, FGF-2, TGF-a, G-CSF, GM-CSF, IFN-γ, IP-10, MCP-1, MIP- 1a, MIP-1b, PDGF-BB, RANTES, TNF-a, VEGF)	Target activation and (patho)physiological response	Patients	[115]
		Hepatocyte growth factor	0/1 (0%)	-	-	-	Patients	[116]
	Small molecule	EAAT2	0/1 (0%)	-	-	-	Patients	[117]
		Putative mitochondrial modulation	0/1 (0%)	-	-	-	HVs	[118]
		Inflammatory macrophages and monocytes regulation	1/1 (100%)	Blood monocyte immune activation markers CD16, HLA-DR	-	Target activation	Patients	[119]
		SOD1	2/2 (100%)	Erythrocyte SOD1 enzymatic activity; Leukocyte actin-normalized SOD1	CSF SOD1 protein and enzymic activity	Target activation	Patients	[120,121]
	Supplement	Lysosomal Cathepsins B and L	0/1 (0%)	-	-	-	Patients	[122]
		Stabilize the mitochondrial transition pore, buffer intracellular energy stores, stimulate synaptic glutamate uptake, and scavenge reactive oxygen species	1/1 (100%)	-	MRS brain glutamate and glutamine (Glx)	Physiological response	Patients	[123]
Overall use of mechanistic biomarkers in early phase ALS trials			14/27 (52%) *					
ATTR amyloidosis	Antisense oligonucleotide	Transthyretin (TTR)	1/1 (100%)	Plasma TTR	-	Target activation	HVs	[124]
	RNA interference	Transthyretin amyloid	1/1 (100%)	Serum transthyretin, retinol-binding protein and vitamin A	-	Target occupancy and activation	HVs and patients	[125]
Overall use of mechanistic biomarkers in early phase ATTR trials			2/2 (100%)					
FRDA	Small molecule	FXN gene expression	1/1 (100%)	Whole blood FXN mRNA, frataxin protein; PBMC chromatin modification via H3 lysine 9 acetylation	-	Target occupancy and activation	Patients	[126]
	Supplement	FXN gene expression	1/1 (100%)	PBMC FXN mRNA and frataxin protein); Blood heterochromatin modifications at the FXN locus	-	Target occupancy and activation	Patients	[127]
	Polyunsaturated fatty acid	Lipid peroxidation	1/1 (100%)	RBC compartment D2-LA	-	Target occupancy	Patients	[128]
Overall use of mechanistic biomarkers in early phase FRDA trials			3/3 (100%)					
FTD	Small molecule	Progranulin protein (PGRN)	1/1 (100%)	Plasma PGRN, PGRN-related inflammatory markers (CRP, ESR), blood cytokines (IL-10, IL-2, IL-6, IL-8, TNFa)	CSF PGRN, NfL, Aβ42, tau, cytokines (IL-10, IL-2, IL-6, IL-8, TNFa); MRI volumetric assessment	Target activation and (patho)physiological response	Patients	[129]
Overall use of mechanistic biomarkers in early phase FTD trials			1/1 (100%)					
GM2 gangliosidosis	Small molecule	β-hexosaminidase (Hex)	1/1 (100%)	Leucocyte and plasma Hex A, β-galactosidase and glucocerebrosidase activity, β-glucuronidase and acid phosphatase	-	Target activation	Patients	[130]
Overall use of mechanistic biomarkers in early phase GM2 gangliosidosis trials			1/1 (100%)					
HD	Antisense oligonucleotide	HTT mRNA	1/1 (100%)	-	CSF mutant HTT, NfL; MRI ventricular volume	Target activation and pathophysiological response	Patients	[131]
	Peptide	Cardiolipin	1/1 (100%)	MRI skeletal muscle dynamic 31P-MRS; PBMC mitochondrial membrane potential (∆Ψm)	MRI brain 31P-MRS; CNS functional domain test battery (NeuroCart^®^)	Target activation and (patho)physiological response	Patients	[132]
Overall use of mechanistic biomarkers in early phase HD trials			2/2 (100%)					
Leber Hereditary Optic Neuropathy	Gene therapy	Mitochondrial gene encoding NADH:ubiquinone oxidoreductase subunit 4 (ND4)	1/2 (50%)	-	OCT average retinal nerve fiber layer (RNFL) thickness; Pattern electroretinogram amplitudes	Physiological response	Patients	[133,134]
Overall use of mechanistic biomarkers in early phase Leber Hereditary Optic Neuropathy trials			1/2 (50%)					
MS	Antibody	Semaphorin 4D	1/1 (100%)	T-cell cSEMA4D expression and saturation; Serum sSEMA4D	-	Target occupancy and activation	Patients	[135]
	Cell therapy	Neurotrophic and immunomodulatory effects, neurogenesis	2/2 (100%)	Lymphocyte subsets (CD4+, CD25+ and CD40+ lymphocytes and CD83+, CD86+, and HLA-DR+ myeloid dendritic cells); PBMC cytokine production	MRI labeled cell localization and volumetric assessment; OCT average retinal nerve fiber layer (RNFL); Vision (HCVA, LCLA)	Target occupancy and (patho)physiological response	Patients	[136,137]
	Small molecule	Anti-inflammatory	1/1 (100%)	PBMC monocyte and 6-sulpho LacNAc + dendritic cell (slanDC) frequency, properties, and activation status	-	Target activation and physiological response	Patients	[138]
		Mitochondrial ATP production (coenzyme Q10)	1/1 (100%)	-	CSF mitochondrial dysfunction markers (GDF15, lactate), NfL, sCD14; BBB leakage (albumin quotient); OCT retinal nerve fiber layer thinning; MRI brain ventricular volume	(patho)physiological response	Patients	[139]
Overall use of mechanistic biomarkers in early phase MS trials			5/5 (100%)					
MSA	Cell therapy	Neurotrophic factors secretion	1/1 (100%)	-	CSF neurotrophic factors (NGF, GDNF, BDNF)	Physiological response	Patients	[140]
	Immunotherapy	α-Synuclein	1/1 (100%)	Serum immunopeptide titers, α-synuclein native epitope titers	-	Target activation	Patients	[141]
Overall use of mechanistic biomarkers in early phase MSA trials			2/2 (100%)					
NCLs	Cell therapy	Palmitoyl-protein thioesterase 1 (PPT-1) and tripeptidyl peptidase 1 (TPP1) enzymes production	0/1 (0%)	-	-	-	Patients	[142]
CLN2 disease	Enzyme replacement	Lysosomal enzyme TPP1	0/1 (0%)	-	-	-	Patients	[143]
Overall use of mechanistic biomarkers in early phase NCLs trials			0/2 (0%)					
NPC1	Cyclodextrin	Neuronal cholesterol homoeostasis	1/1 (100%)	Serum a24(S)-hydroxycholesterol (24[S]-HC)	CSF a24(S)-hydroxycholesterol (24[S]-HC), fatty acid binding protein 3 (FABP3) and calbindin D19	Target activation and (patho)physiological response	Patients	[144]
Overall use of mechanistic biomarkers in early phase NPC1 trials			1/1 (100%)					
PD	Antibody	α-synuclein	3/3 (100%)	Plasma antibody/α-syn complexes; Serum total and free α-synuclein	CSF total and free α-synuclein, total Aβ, Aβ42, DJ-1, DAT scan	Target occupancy, activation, and pathophysiological response	HVs and patients	[145,146,147]
	Cell therapy	Neurotrophic factors to restore dopaminergic cell function	0/1 (0%)	-	-	-	Patients	[148]
	Gene therapy	Aromatic L-amino acid decarboxylase (AADC)	3/3 (100%)	-	PET FMT brain AADC expression and activity	Target occupancy and activation	Patients	[149,150,151]
		Tyrosine hydroxylase, AADC, cyclohydrolase 1	1/1 (100%)	-	PET cortical excitability and reflex recordings	Physiological response	Patients	[152]
	Growth factor	Granulocyte colony-stimulating factor (G-CSF)	0/1 (0%)	-	PET 18 F-DOPA for disease progression	Pathophysiological response	Patients	[153]
		Granulocyte-macrophage colony-stimulating factor (GM-CSF)	1/1 (100%)	Expression of Treg phenotype and function (CD4+ Teffs (CD4+CD127hiCD25hi), CD4+ Tregs (CD4+CD127loCD25hi), FOXP3+CD4+ Tregs, iCTLA4+CD4+ Tregs, CD39+CD4+ Tregs, and f FAS+CD4+ Tregs), T cell proliferation mRNA (GATA4, IL2, HOXA10, and KIF2C), anti-inflammatory gene expression (PPARG, LRRC32, FOSL1, IL1R2, IL13RA1, NR4A3, GFI1), tryptophan pathway targeted metabolomics	-	Target activation and physiological response	Patients	[154]
		rhPDGF-BB (proliferation of SOX-2/Olig-1– positive periventricular progenitor cells)	1/1 (100%)	-	[11C]PE2I DAT binding	Pathophysiological response	Patients	[155]
	Immunotherapy	α-Synuclein	1/1 (100%)	Serum antibody titres	CSF antibody titres, total α-synuclein, Aβ1–42, p-tau	Target activation and pathophysiological response	Patients	[156]
	Deep brain stimulation	Unknown	0/1 (0%)	-	-	N/A	Patients	[157]
	Small molecule	Glucosylceramide synthase (GCS)	1/1 (100%)	Plasma glucosylceramide (GL-1), globostriaosylceramide (GL-3), and GM3 ganglioside (GM3)	-	Target activation	HVs	[158]
		Myeloperoxidase	1/1 (100%)	-	PET distribution volume of 11C-PBR28 binding to microglia marker TSPO	Target occupancy	Patients	[159]
		Flavonoid (regulating dopaminergic system function, anti-oxidative damage and anti-inflammatory effects)	0/1 (0%)	-	-	N/A	HVs	[160]
	Supplement	Antioxidant	0/1 (0%)	-	-	N/A	Patients	[161]
Overall use of mechanistic biomarkers in early phase PD trials			12/17 (71%)					
PSP	Antibody	Tau protein	0/2 (0%)	-	-	N/A	Patients	[31,162]
	Cell therapy	Trophic, anti-apoptotic and regenerative effects	0/1 (0%)	-	MRI, SPECT and PET with tropanic tracers (FP-CIT and Beta-CIT) longitudinal neuroimaging	Pathophysiological response	Patients	[163]
	Small molecule/Blood product	Acetylation of tau/unknown	1/1 (100%)	Plasma NfL concentrations	CSF amyloid beta Aβ, t-tau, p-tau181; MRI brain volumetric assessment	(patho)physiological response	Patients	[164]
Overall use of mechanistic biomarkers in early phase PSP trials			1/4 (25%)					
SCA	Cell therapy	Trophic factor secretion, immunomodulation	1/1 (100%)	-	PET brain glucose metabolism	Physiological response	Patients	[165]
	Growth factor	Antiapoptotic, antioxidative, anti-inflammatory, neurotrophic and angio- genic properties	0/1 (0%)	-	-	N/A	HVs	[166]
Overall use of mechanistic biomarkers in early phase SCA trials			1/2 (50%)					
SMA	Antisense oligonucleotide	SMN2 mRNA splicing	1/1 (100%)	-	CSF SMN protein	Target activation	Patients	[167]
	Small molecule	SMN2 splicing	2/2 (100%)	Blood mRNA (full-length SMN2, SMN1, SMNΔ7), SMN protein	-	Target activation	HVs and patients	[168,169]
	Gene therapy	SMN	0/1 (0%)	-	-	N/A	Patients	[170]
Overall use of mechanistic biomarkers in early phase SMA trials			3/4 (25%)					

Abbreviations: AADC = aromatic L-amino acid decarboxylase; Aβ = amyloid β; AChE = Acetylcholinesterase; AD = Alzheimer’s disease; ALS = amyotrophic lateral sclerosis; AAP = amyloid precursor protein; ATP = adenosine triphosphate; ATTR = amyloid transthyretin; BACE1 = beta-secretase 1; BBB = blood-brain barrier; BDNF = brain-derived neurotrophic factor; Beta-CIT = 18F-Fluoro-2-deoxyglucose labeled tropanic SPECT tracer;; BMC = bone marrow concentrated cells; CD# = cluster of differentiation #; ChAT = choline acetyltransferase; CLN2 = classic late infantile neuronal ceroid lipofuscinosis; CMAP = compound muscle action potential; CRP = C-reactive protein; cSEMA4D = T-cell semaphorin 4D; CSF = cerebrospinal fluid; CTLA4 = cytotoxic T-lymphocyte-associated protein 4; DAT = dopamine active transporter; DBS = deep brain stimulation; DJ-1 = protein deglycase DJ-1 (PARK7); D2-LA = di-deutero isotopologue of linoleic acid ethyl ester; EAAT2 = excitatory amino acid transporter 2; EIM = electrical impedance myography; EMG = electromyogram; ESR = erythrocyte sedimentation rate; ET(B) = endothelin receptor type B; FABP3 = fatty acid binding protein 3; FD = fiber density; FDG = fluorine-18-deoxyglucose; FGF-# = fibroblast growth factor #; FMT = [18F] fluorometatyrosine; FOSL1 = FOS like 1, AP-1 transcription factor subunit; FOXP3 = forkhead box P3; FRDA = Friedreich ataxia; FTD = frontotemporal dementia; FP-CIT = [^123^I] labeled tropanic SPECT tracer; FXN = frataxin; GATA4 = transcription factor GATA-4; GCS = glucosylceramide synthase; GDF15 = growth/differentiation factor 15; GDNF = glial cell-derived neurotrophic factor; GFAP = glial fibrillary acidic protein; GFI1 = growth factor independent 1 transcriptional repressor; Glcr = β-glucuronidase; GL-1 = glucosylceramide; GL-3 = globotriasylceramide; Glx = glutamate and glutamine; GM3 = monosialodihexosylganglioside; GM-CSF = granulocyte-macrophage colony-stimulating factor; GS = glycogen synthase; GSK3β = glycogen synthase kinase-3β; G-CSF = granulocyte colony-stimulating factor; HCVA = high-contrast visual acuity; HD = Huntington’s disease; Hex = β-hexosaminidase; HGF = Hepatocyte growth factor; HLA-DR = human leukocyte antigen DR; HOXA10 = homeobox A10; HTT = huntingtin; HVs = healthy volunteers; IFN-γ = interferon gamma; IgG = immunoglobulin G; IgM = immunoglobulin M; IL-# = Interleukin #; IP-10 = interferon gamma-induced protein 10; KIF2C = kinesin Family Member 2C; KLH = keyhole limpet hemocyanin; LacNAc = N-acetyllactosamine; LCLA = low-contrast letter acuity; LRRC32 = leucine rich repeat containing 32; MCP-1 = monocyte chemoattractant protein 1; MIP-# = macrophage inflammatory protein #; MRI = magnetic resonance imaging; mRNA = messenger RNA; MRS = magnetic resonance spectroscopy; MS = multiple sclerosis; MSA = multiple system atrophy; MUNE = motor unit number estimation; MUNIX = motor unit number; MUSIX = motor unit size; NADH = nicotinamide adenine dinucleotide; NCLs = neuronal ceroid lipofuscinoses; ND4 = NADH-ubiquinone oxidoreductase chain 4; NGF = nerve growth factor; NfL = neurofilament light chain; NPC1 = Niemann-Pick disease type C1; NR4A3 = nuclear receptor subfamily 4 group A member 3; OCT = oOptical coherence tomography; PBMCs = peripheral blood mononuclear cells; PD = Parkinson’s disease; PDGF-BB = platelet-derived growth factor BB; PET = positron emission tomography; PGRN = progranulin protein; PiB = Pittsburgh compound B; PPARG = peroxisome proliferator-activated receptor gamma; PPT-1 = palmitoyl-protein thioesterase 1; PSP = progressive supranuclear palsy; pS166 = phosphorylation of serine 166; p-NfH = phosphorylated neurofilament heavy chain; p-tau181 = tau phosphorylated at threonine 181; QC = glutaminyl cyclase; RANTES = regulated on activation, normal T cell expressed and secreted; RBC = red blood cells; rhPDGF-BB = recombinant human platelet-derived growth factor-BB; RIPK1 = receptor-interacting serine/threonine-protein kinase 1; RNA = ribonucleic acid;RNFL = retinal nerve fiber layer; sAPP = soluble amyloid precursor protein; SCA = spinocerebellar ataxia; sCD14 = soluble CD14; sIL-2r = soluble IL-2 receptor; slanDCs = 6-sulfo; LacNAc dendritic cells; SMA = spinal muscular atrophy; SMN# = survival of motor neuron #; SMNΔ7 = exon 7-deleted SMN protein; SMUP = single motor unit potential; SOD1 = superoxide dismutase 1; SOX-2/Olig-1 = SRY-box transcription factor 2/oligodendrocyte transcription factor 1; SPECT = single photon emission computed tomography; sSEMA4D = soluble semaphorin 4D; TA = tibialis anterior; Teffs = effector T cells; TGF-# = transforming growth factor #; TNF-α = tumor necrosis factor; TPP1 = tripeptidyl peptidase 1; Tregs = regulatory T cells; TSPO = translocator protein; TTR = transthyretin; t-tau = total tau; VEGF = vascular endothelial growth factor; YKL-40 = chitinase-3-like-1 protein; 24[S]-HC = a24(S)-hydroxycholesterol; 31P-MRS = 31P-magnetic resonance spectroscopy; 5-HT2A = 5-hydroxy-tryptamine 2A; ΔΨm = mitochondrial membrane potential; [11C]PE21 = selective dopamine active transporter (DAT) radiotracer; [11C]-PBR28 = 18pkD translocator protein (TSPO) radiotracer. * RIPK1 was under development for multiple indications (AD and ALS) in healthy subjects and has been added to the totals for both indications. AD is listed only once in the table to avoid duplication.

## Data Availability

Not applicable.

## Supplementary Materials

The following are available online at https://www.mdpi.com/1422-0067/22/4/1615/s1.
