# Supplementary material for: Targeting for Success: Demonstrating Proof-of-Concept with Mechanistic Early Phase Clinical Pharmacology Studies for Disease-Modification in Neurodegenerative Disorders
Source: Int J Mol Sci. 2021 Feb 5;22(4):1615. doi: 10.3390/ijms22041615 (PMC7915613; doi:10.3390/ijms22041615)

Pubmed review search strategy

The following MESH search strategy was used in PubMed for initial article selection for this review.

(("Neurodegenerative Diseases"[Mesh] OR "Degenerative Neurologic Condition"[tw] OR "Degenerative Neurologic Conditions"[tw] OR "Degenerative Neurologic Disease"[tw] OR "Degenerative Neurologic Diseases"[tw] OR "Degenerative Neurologic Disorder"[tw] OR "Degenerative Neurologic Disorders"[tw] OR "Degenerative Neurological Condition"[tw] OR "Degenerative Neurological Conditions"[tw] OR "Degenerative Neurological Disease"[tw] OR "Degenerative Neurological Diseases"[tw] OR "Degenerative Neurological Disorder"[tw] OR "Degenerative Neurological Disorders"[tw] OR "Nervous System Degenerative Conditions"[tw] OR "Nervous System Degenerative Disease"[tw] OR "Nervous System Degenerative Diseases"[tw] OR "Nervous System Degenerative Disorders"[tw] OR "Neurodegenerative Condition"[tw] OR "Neurodegenerative Conditions"[tw] OR "Neurodegenerative Disease"[tw] OR "Neurodegenerative Diseases"[tw] OR "Neurodegenerative Disorder"[tw] OR "Neurodegenerative Disorders"[tw] OR "Neurologic Degenerative Condition"[tw] OR "Neurologic Degenerative Disease"[tw] OR "Neurologic Degenerative Diseases"[tw] OR "Neurologic Degenerative Disorder"[tw] OR "Neurologic Degenerative Disorders"[tw] OR "Neurological Degenerative Conditions"[tw] OR "Neurological Degenerative Disease"[tw] OR "Neurological Degenerative Diseases"[tw] OR "Neurological Degenerative Disorder"[tw] OR "Neurological Degenerative Disorders"[tw] OR "Degenerative Neurologic"[tw] OR "Degenerative Neurological"[tw] OR "Nervous System Degenerative"[tw] OR "Neurodegenerative"[tw] OR "Neurologic Degenerative"[tw] OR "Neurological Degenerative"[tw] OR "Alexander Disease"[tw] OR "Alstrom Syndrome"[tw] OR "Alzheimer Disease"[tw] OR "Alzheimers Disease"[tw] OR "Alzheimer's Disease"[tw] OR "Amyotrophic Lateral Sclerosis"[tw] OR "Anti-N-Methyl-D-Aspartate Receptor Encephalitis"[tw] OR "Autosomal Dominant Optic Atrophy"[tw] OR "Bovine Spongiform Encephalopathy "[tw] OR "Bovine Spongiform Encephalopathies"[tw] OR "Canavan Disease"[tw] OR "Charcot-Marie-Tooth Disease"[tw] OR "Chronic Traumatic

Encephalopathies"[tw] OR "Chronic Traumatic Encephalopathy"[tw] OR "Chronic Wasting Disease"[tw] OR "Cockayne Syndrome"[tw] OR "Diffuse Neurofibrillary Tangles with Calcification"[tw] OR "Dystonia Musculorum Deformans"[tw] OR "Familial Amyloid Neuropathies"[tw] OR "Familial Amyloid Neuropathy"[tw] OR "Familial Dysautonomia"[tw] OR "Familial Optic Atrophy"[tw] OR "Familial Spastic Paraplegia"[tw] OR "Fatal Familial Insomnia"[tw] OR "Friedreich Ataxia"[tw] OR "Friedreich Ataxias"[tw] OR "Frontotemporal Dementia"[tw] OR "Frontotemporal Lobar Degeneration"[tw] OR "Gerstmann-Straussler-Scheinker Disease"[tw] OR "Giant Axonal Neuropathies"[tw] OR "Giant Axonal Neuropathy"[tw] OR "Hepatolenticular Degeneration"[tw] OR "Hereditary Central Nervous System Demyelinating"[tw] OR "Hereditary Central Nervous System Demyelinating Diseases"[tw] OR "Hereditary Sensory and Autonomic Neuropathy"[tw] OR "Hereditary Sensory and Motor Neuropathy"[tw] OR "Leber Optic Atrophy"[tw] OR "Huntington Disease"[tw] OR "Kuru"[tw] OR "Lafora Disease"[tw] OR "Lambert-Eaton Myasthenic Syndrome"[tw] OR "Lewy Body Disease"[tw] OR "Limbic Encephalitis"[tw] OR "Machado-Joseph Disease"[tw] OR "Motor Neuron Disease"[tw] OR "Motor Neuron Diseases"[tw] OR "Multiple System Atrophies"[tw] OR "Multiple System Atrophy"[tw] OR "Myasthenia Gravis"[tw] OR "Myoclonic Cerebellar Dyssynergia"[tw] OR "Myotonia Congenita"[tw] OR "Myotonic Dystrophies"[tw] OR "Myotonic Dystrophy"[tw] OR "Nervous System Heredodegenerative"[tw] OR "Nervous System Heredodegenerative Disorders"[tw] OR "Nervous System Paraneoplastic Syndromes"[tw] OR "Neurofibromatoses"[tw] OR "Neurofibromatosis"[tw] OR "Neuronal Ceroid-Lipofuscinoses"[tw] OR "Olivopontocerebellar Atrophies"[tw] OR "Olivopontocerebellar Atrophy"[tw] OR "Opsoclonus-Myoclonus Syndrome"[tw] OR "Pantothenate Kinase-Associated Neurodegeneration"[tw] OR "Paraneoplastic Cerebellar Degeneration"[tw] OR "Paraneoplastic Polyneuropathies"[tw] OR "Paraneoplastic Polyneuropathy"[tw] OR "Parkinson Disease"[tw] OR "Parkinsons Disease"[tw] OR "Parkinson's Disease"[tw] OR "Postpoliomyelitis Syndrome"[tw] OR "Primary Progressive Nonfluent Aphasia"[tw] OR "Prion Disease"[tw] OR "Prion Diseases"[tw] OR "Progressive Bulbar Palsy"[tw] OR "Progressive Supranuclear Palsies"[tw]

OR "Progressive Supranuclear Palsy"[tw] OR "Refsum Disease"[tw] OR "Scrapie"[tw] OR "Spinal Muscular Atrophies"[tw] OR "Spinal Muscular Atrophy"[tw] OR "Spinocerebellar Ataxia"[tw] OR "Spinocerebellar Ataxias"[tw] OR "Spinocerebellar Degeneration"[tw] OR "Spinocerebellar Degenerations"[tw] OR "Subacute Combined Degeneration"[tw] OR "Synucleinopathies"[tw] OR "Synucleinopathy"[tw] OR "Tauopathies"[tw] OR "Tauopathy"[tw] OR "TDP-43 Proteinopathies"[tw] OR "TDP-43 Proteinopathy"[tw] OR "Tourette Syndrome"[tw] OR "Transverse Myelitis"[tw] OR "Tuberous Sclerosis"[tw] OR "Unverricht-Lundborg Syndrome"[tw] OR "Wolfram Syndrome"[tw]) AND ("Clinical Trial, Phase I"[pt] OR "Microdosing Trial"[tw] OR "Human Microdosing Trials"[tw] OR "Phase 1 Clinical Trial"[tw] OR "Phase 1 Clinical Trials"[tw] OR "Phase i Clinical Trial"[tw] OR "Phase i Clinical Trials"[tw] OR "Phase One Clinical Trial"[tw] OR "Phase One Clinical Trials"[tw] OR "Phase 1"[tw] OR "Phase i"[tw] OR "Phase One"[tw] OR "Clinical Trials, Phase I as Topic"[Mesh]) AND ("2010/01/01"[PDAT] : "3000/12/31"[PDAT]))

Figure S1. Study selection overview

Flow diagram of studies' screening and selection for this review.

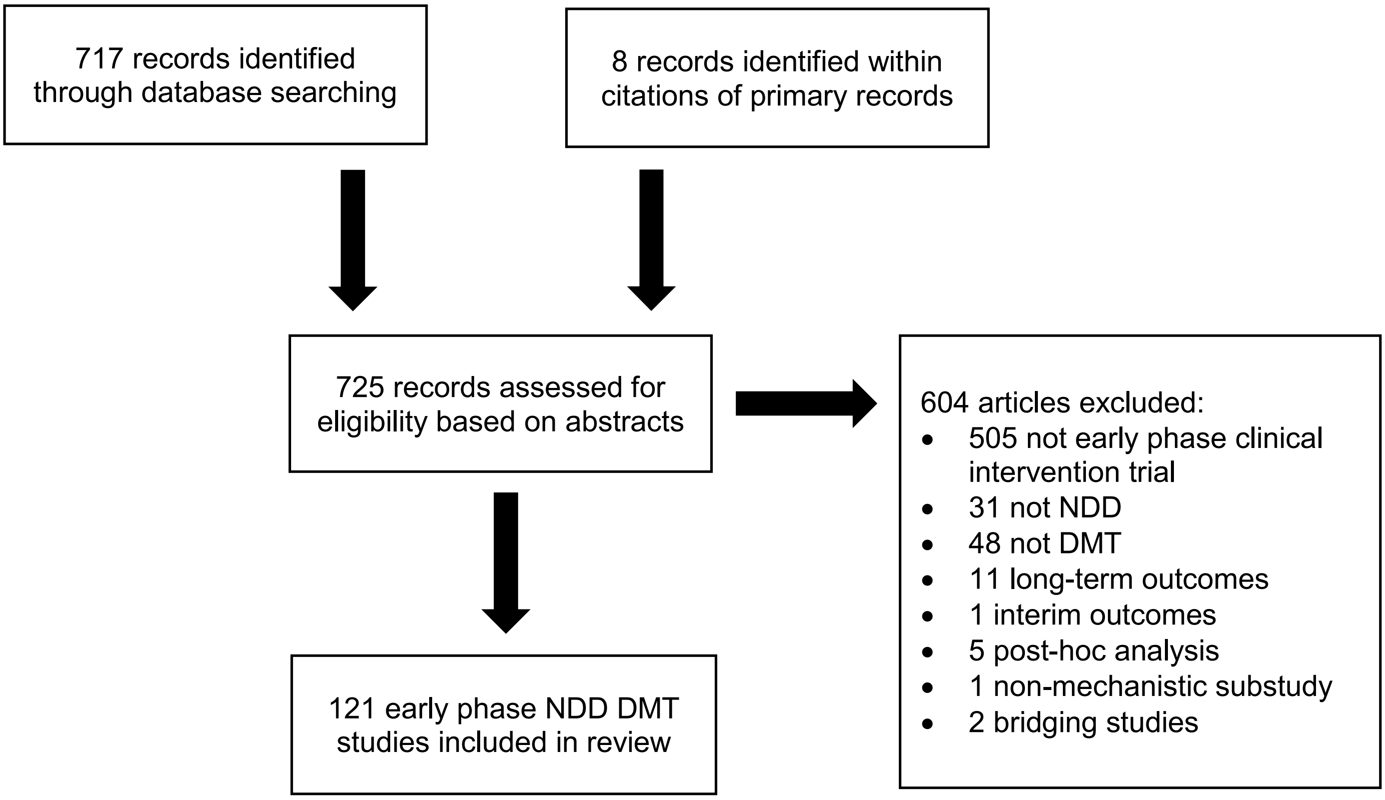

Supplement: Supplementary file 1 [file ijms-22-01615-s001.pdf]
